# Path analysis of influencing factors for maternal antenatal depression in the third trimester

**DOI:** 10.1038/s41598-024-55355-4

**Published:** 2024-02-27

**Authors:** Yanchi Wang, Jian Gu, Feng Zhang, Xujuan Xu

**Affiliations:** 1 Affiliated Nantong Hospital of Shanghai University (The Sixth People’s Hospital of Nantong), Nantong, Jiangsu China; 2 Medical School of Nantong University, Nantong, Jiangsu China; 3 Department of Nursing, Affiliated Hospital of Nantong University, Nantong, 226001 Jiangsu China; 4 Department of Epidemiology and Medical Statistics, School of Public Health, Nantong University, Nantong, Jiangsu China; 5 Medical School (School of Nursing), Nantong University, Nantong, 226001 Jiangsu China

**Keywords:** Maternal, Antenatal depression, Influencing factors, Path analysis, Psychology, Risk factors, Signs and symptoms

## Abstract

Maternal antenatal depression (AD) is a nonpsychotic depressive episode during pregnancy that can harm both the pregnant woman and the fetus. This study aimed to investigate the intrinsic interrelationships between AD and its influencing factors by constructing a path model. This survey-based cross-sectional study included 1071 pregnant women who underwent pregnancy examinations in three hospitals in Nantong City, China, between February and June 2023. General information and information regarding maternal AD, pregnancy stress, prenatal anxiety, social support, marital satisfaction, sleep quality, and resilience were collected. Multiple linear regression analysis using SPSS 25.0 was employed to determine the factors influencing pregnancy depression, and Amos25.0 was used to construct a structural equation model. AD incidence was 19.4% (208/1071). The independent risk factors affecting AD in pregnant women have been integrated into the established path analysis model. The model demonstrated a good fit (χ^2^/DF = 1.238, comparative fit index = 0.999, goodness-of-fit index = 0.998, normed fit index = 0.996, adjusted goodness-of-fit index = 0.990, incremental fit index = 0.999, and root mean square error of approximation = 0.015). While prenatal anxiety (0.230) and hyperthyroidism (0.048) only had direct effects on AD, mental resilience was the biggest factor affecting AD, followed by pregnancy stress, marital satisfaction, prenatal anxiety, sleep quality, social support, and hyperthyroidism. Improved mental resilience, social support, sleep quality, and marital satisfaction; reduced pregnancy stress and prenatal anxiety; and effective hyperthyroidism treatment might reduce AD. This study underscored the significance of delivering actionable strategies and tangible assistance to pregnant women to reduce AD.

## Introduction

Maternal antenatal depression (AD) is a type of depressive episode that is not accompanied by psychosis and occurs during pregnancy. It manifests as low mood, reduced concentration, sleeping and eating difficulties, as well as persistent feelings of irritability and anxiety^1^. According to a systematic review, the pooled prevalence of AD in high-income countries was 9.2% but doubled to as high as 19.2% in low- and middle-income countries^2^. AD has negative impacts on both the well-being of the mother and the development of the fetus/child. These repercussions include an increased risk of preterm births, low birth weights in infants, preeclampsia, as well as severe emotional and behavioral complications such as suicidal tendencies^3^. AD serves as an indicator for postpartum depression, as nearly half of the instances of postpartum depression occur during pregnancy^4^. Reportedly, the offsprings of women with AD are at a heightened risk of depression during adolescence^5^. However, despite the seriousness of AD, only approximately 50% of women with AD are identified and treated. Compared to prenatal anxiety, antenatal depression is more likely to have serious consequences for women and their fetuses, therefore, in this study, antenatal depression is used as the outcome variable. Therefore, women with AD require heightened attention, and implementing effective treatment interventions is essential for providing quality prenatal care.

Reportedly, AD prevalence is higher in Asian populations (24.3%)^6^. Studies conducted in China showed that the prevalence of depression during pregnancy was 15–30%^7,8^. Furthermore, considering research involving Chinese pregnant women from different trimesters, with diverse demo-socioeconomic backgrounds, and utilizing various study methodologies, the prevalence rate of AD ranged from 4 to 46.11%^9,10^. According to a meta-analysis, the prevalence of AD is generally greater during the second and third trimesters of pregnancy (12.4%), in comparison to the first trimester (7.4%)^11^. However, another study found that the prevalence of AD was elevated during the third trimester in contrast to both the first and second trimesters of pregnancy^9^. Elevated depressive symptoms during the third trimester were linked to diminished neonatal brain functional connectivity in the frontal lobe and between the frontal/temporal and occipital lobes^12^. This underscores the significance of maternal depressive symptoms on the developmental trajectory of the offspring's brain, even in the absence of clinical depression. The prevalence of AD is not fixed. It varies from country to country and region to region. China is a middle-income country, a systematic review indicated that prevalence of AD was 9.2 in high-income countries and 9.5 in low-and middle-income countries. A recent systematic review noted that AD is common in low-and middle-income countries, affecting one in four perinatal women. Prevalence was highest in low-and middle-income countries, with a total prevalence of 25.5%^13^. A systematic review and meta-analysis of perinatal depression in Chinese mainland showed that the prevalence of AD was 19.7%^14^. Therefore, it is of utmost importance to ascertain the factors contributing to depression among Chinese pregnant women during the third trimester of pregnancy.

A review suggested that the main psychological risk factors for AD were prenatal anxiety and pregnancy stress^15^, affecting about 17% and as high as 84% of women, respectively^16^. A study conducted in China revealed that over 50% of pregnant women experience symptoms of anxiety and stress, particularly during the later stages of pregnancy. Stress may increase anxiety and disrupt sleep quality, thereby increasing the likelihood of AD. Anxiety can increase individuals' sensitivity to stress, thereby increasing the risk of antenatal depression^17^. Reportedly, some of the most common risk factors for AD are social support and marital satisfaction^18^. Social support can provide emotional support and resources, mitigating the pressure faced by pregnant women and reducing the likelihood of AD^19^. Higher levels of marital satisfaction are associated with a lower risk of AD. Stable and satisfying marital relationships can provide emotional support and resource sharing, reducing psychological stress and AD for pregnant women^20^. Meanwhile, poor sleep quality is associated with a higher risk of AD. Decreased sleep quality can lead to physical and emotional discomfort, increasing the occurrence and severity of AD^21^. According to a previous study^22^, the quality of sleep deteriorates as the gestational age of the pregnant woman advances. The FinnBrain Birth Cohort Study^23^ reveals that sleep disturbances have an impact on depressive symptoms specifically during the third trimester, which is consistent with the findings of Okun et al.'s study^24^. In addition, inadequate psychological resilience can elevate the likelihood of depression among pregnant women^25^. Prior studies have indicated that resilience plays a safeguarding role against psychological distress during pregnancy. Individuals with higher resilience are better able to adapt to life stress and adversity, thereby reducing the risk of AD. Resilience can protect against antenatal depression by alleviating stress, promoting positive emotions, and facilitating adaptive coping strategies^8,26,27^.

Social support, resilience, and marital satisfaction may interact with each other, reducing stress and enhancing coping ability through emotional support and resource sharing, thus lowering the risk of AD^28^. High levels of stress may have a negative impact on sleep quality, leading to a decrease in sleep quality and an increased risk of AD^29^. Social support, resilience, stress, and sleep quality play important roles in influencing prenatal anxiety^25,30,31^. The following is an explanation of the basic principles between them: Social support refers to emotional support, informational support, and tangible assistance from family, friends, and the community. Studies have shown that pregnant women who lack social support are more likely to experience anxiety symptoms. Social support can help alleviate stress, provide emotional comfort, and offer effective problem-solving strategies, thereby reducing the risk of prenatal anxiety. Resilience is the ability to cope with stress and adversity. Individuals with higher psychological resilience are better equipped to deal with stress, thus reducing the occurrence of anxiety and depression. For pregnant women, psychological resilience can help them better adapt and cope with physical and emotional changes, thus minimizing the occurrence of anxiety symptoms. Various sources of stress during pregnancy, such as family pressure and life changes, can increase the risk of prenatal anxiety. The greater the stress faced by pregnant women, the higher the likelihood of prenatal anxiety. Sleep quality has a significant impact on prenatal anxiety. Pregnant women may experience sleep disturbances, such as insomnia and increased nocturnal awakenings, during pregnancy. Poor sleep quality increases the risk of anxiety and emotional instability.

The impact of AD on maternal health and postpartum health has been studied and it has been shown that AD is associated with physical and mental health issues in pregnant women. Symptoms of depression during pregnancy can have negative effects on maternal-infant relationships, fetal health, and the risk of postpartum depression. The factors influencing antenatal depression in pregnant women include sleep quality, marital satisfaction, social support, resilience, pregnancy stress, and anxiety, among others. Most of the literature has primarily investigated one of these factors, rarely discussing all these factors within a single model.

Social support, resilience, and marital satisfaction are three intrinsic and difficult-to-change variables that develop during pregnancy. Therefore, these three variables serve as the starting variables in the path. The variables of social support, resilience, and marital satisfaction all have an impact on pregnancy stress, so these three variables point towards pregnancy stress. Pregnancy stress, in turn, affects the quality of sleep during pregnancy. The influence of anxiety on depression is particularly strong, therefore the path from pregnancy stress points towards sleep, and sleep points towards anxiety. We construct this path analysis based on the above assumptions.

This paper integrates common variables related to AD in pregnant women. By providing clear explanations of the nature, magnitude, and significance of path coefficients, we can better understand the relationships between variables in the model. Path coefficients are numerical values that represent direct and indirect relationships between variables in structural equation models. By interpreting path coefficients, we can understand how variables interact with each other and contribute to the overall explanatory power of the model. While the variables used in the study may not produce new findings, they may provide a deeper and more comprehensive understanding, and offer more evidence to support research in this field. Further analysis and interpretation of the logical explanations of the research intent may be required based on the specific content and conclusions of this paper. For instance, a recent study found that pregnancy stress had an indirect effect on AD through a mediated variable-resilience^32^. These findings suggest that neglecting the interplay among variables might hinder our ability to identify potential risk factors for AD, while traditional analytical methods may not fully reveal their interrelationships. Thus, the main objective of this study was to employ a path model to uncover the risk factors and their interconnections in order to facilitate the implementation of effective interventions and control the rising incidence of AD.

## Methods

### Design and participants

This cross-sectional study included pregnant women who underwent pregnancy examination in the Department of Obstetrics and Gynecology of three tertiary hospitals in Nantong City, China, between February and June 2023. We employed convenience sampling techniques to randomly distribute questionnaires to women who attended for pregnancy check-ups on the same day, ensuring an equal likelihood of selection for each pregnant woman. The data from the questionnaires was collected and entered SPSS Windows version 25.0 for analysis. The inclusion criteria were as follows: (1) maternal age ≥ 18 years and (2) participants who can cooperate with the survey, communicate effectively, understand the content of the questionnaire, and complete the questionnaire independently. The exclusion criteria were as follows: (1) pregnant women who have been clinically identified with mental health issues, such as schizophrenia, mania, and bipolar disorder; (2) pregnancies with abnormalities like fetal deformities; (3) a past history of mental illness and cognitive dysfunction.

During the hospital visits, trained investigators asked the pregnant women if they wanted to participate in the study, which was anonymous, confidential, and voluntary. Before their involvement in the study, all participants signed an informed consent form and were reminded of their right to withdraw at any stage of the study. Investigators administered in-person questionnaire surveys to women who satisfied the eligibility and exclusion criteria. Any questionnaires that were incomplete were excluded from the analysis. Overall, 1088 pregnant women were surveyed, of which 5 dropped out, 10 had missing data, and 2 provided invalid answers. Finally, 1071 valid questionnaires were included for the analysis, with a survey response rate of 98.44%. The study was approved by the ethics committee of the Affiliated Hospital of Nantong University (approval number: 2022-K150-01). All participants signed an informed consent form voluntarily before participating in the study.

### Measurement

#### Basic characteristics

A baseline questionnaire designed by our research team members was administered to the participants. The questionnaire included the following: (a) basic information such as age, maternal and paternal education, years of marriage, monthly family income, and (b) perinatal conditions such as abortion history, parity, pregnancy complications, assisted reproduction, urinary incontinence, and SARS-CoV-2 infection during pregnancy.

#### Edinburgh Postnatal Depression Scale (EPDS)

EPDS, developed by Cox et al.^33^, was used to assess self-reported depressive symptoms of the participants. The Mainland Chinese version of the EPDS has been validated by Wang et al. and may be applied for the screening of postpartum depression and AD^34^. EPDS consists of 10 items, with each item being scored on a 4-point scale ranging from 0 to 3, and the total score falls within the range of 0–30. Higher scores signify higher likelihood of depression^35^. The Cronbach’s alpha was 0.78, and the test–retest reliability was 0.90^36^. In the Chinese version of the EPDS, a cutoff score of 10 is considered valid^36^.

#### Pregnancy Pressure Scale (PPS)

PPS, which was developed by Chen et al.^37^ in Taiwan, is a self-report assessment tool. It consists of 30 items that are rated on a 4-point scale (0 = not at all, 1 = mild, 2 = moderate, and 3 = severe). PPS was designed to reflect a Chinese cultural framework. This instrument assesses stress related to the health and safety of both the mother and child, recognition of parental roles, and changes in body shape and physical activity. Higher scores indicate higher levels of pregnancy stress. The PPS has demonstrated acceptable reliability among Chinese women^38^. In this study, the PPS was used to measure antenatal stress, and Cronbach’s alpha was 0.94.

#### Depression Anxiety Stress Scale (DASS-21)

DASS-21 is used to assess an individual's negative mood and the severity of symptoms experienced within the previous week^39^. This scale comprises three subscales: anxiety, depression, and stress. Each subscale consists of seven items, resulting in a total of 21 items. Each item is rated on a four-point scale ranging from 0 to 3, representing “completely inconsistent,” “partially consistent,” “mostly consistent,” and “completely consistent.” Higher scores indicated greater intensity of negative emotions. However, in this study, only the anxiety dimension was used. The Cronbach’s alpha for the anxiety dimension was 0.879^40^.

#### Perceived Social Support Scale (PSSS)

The Chinese version of the PSSS, which was developed by Zimet^41^, is used to assess PSS. This scale consists of 12 items that evaluate individuals' perceptions of social support from their families (4 items), friends (4 items), and significant others (4 items). Each item is rated on a 7-point Likert scale, ranging from 1 (very strongly disagree) to 7 (very strongly agree), with higher scores indicating greater perceived social support. The good psychometric characteristics of this scale have been confirmed in the Chinese population^42^. The Cronbach’s alpha was 0.88, and the test–retest reliability was 0.85.

#### Pittsburgh sleep quality index (PSQI)

PSQI^43^ is a self-report questionnaire consisting of 19 items that assess seven dimensions related to sleep quality. The PSQI has been psychometrically evaluated in the Chinese population^44^. The Chinese version of the PSQI has shown satisfactory reliability, with Cronbach's alpha coefficients ranging from 0.77 to 0.84^45^. The total score ranges from 0–21, with higher scores indicating poorer sleep quality. Previous study defined poor sleep quality as a sum score of ≥ 5^46^.

#### Marital Satisfaction Scale (MSS)

MSS^47^ contains 10 items and measures marital satisfaction. Using the 5-point Likert scale, the scores range from “5 = I quite agree with” to “1 = I quite disagree with.” Questions number Q1, Q3, Q5, Q8, and Q9 were negative items and need reversing. The total scoring of this questionnaire ranges from 10 to 50, with a higher score indicating higher marital satisfaction.

#### 10-item Connor-Davidson Resilience Scale (CD-RISC-10)

CD-RISC-10 was a scale consisting of 10 items, co-developed by Connor and Davidson^48^. It has demonstrated good reliability and construct validity, with a Cronbach's alpha coefficient of 0.85. In this study, the Chinese version of the CD-RISC-10, translated and revised by Chinese scholars, was used. The Chinese version showed good psychometric properties, internal consistency, consequential validity, and criterion-related validity, with a Cronbach's alpha coefficient of 0.92^49^. This scale has been used in a study involving Chinese pregnant women^30^. Each item on the CD-RISC-10 is rated on a 5-point Likert scale, with scores ranging from 0 to 4, corresponding to the responses "never," "seldom," "sometimes," "frequently," and "always." The CD-RISC-10 score is calculated as the sum of all item scores. A higher CD-RISC-10 score indicates greater resilience.

### Statistical methods

All variables entered into the path analysis were from multiple linear regressions. Multiple linear regression analysis needs to be conducted because all variables are continuous variables. First, a univariate analysis is performed to identify statistically significant variables (*P* < 0.05). The significant variables are then included in the multiple factor analysis and the significant variables from the multiple factor analysis are included in the model for path model building. We hope to use path analysis to explain the paths of influence between independent variables and the outcome variable. Path analysis is a statistical method used to analyze linear relationships between variables. It involves constructing a structural equation model to uncover the direct and indirect paths through which independent variables influence dependent variables. In path analysis, the relationships between variables are represented by path coefficients, which denote the linear associations between variables. Path analysis assumes that the relationships between variables are linear, meaning that the effects of variables are transmitted through linear pathways. Through path analysis, we can gain insights into the strength and direction of relationships between different variables and evaluate the statistical significance of each path. By providing a clear explanation of the path coefficients in relation to their nature, magnitude, and significance, we can enhance our comprehension of the relationships between variables in the model. Nature of relationships: Positive path coefficients: A positive path coefficient indicates a direct positive relationship between variables. An increase in the independent variable leads to an increase in the dependent variable. Negative path coefficients: A negative path coefficient indicates a direct negative relationship between variables.

In path analysis, CFI (Comparative Fit Index): A comparative fit index used to evaluate model fit. GFI (Goodness-of-Fit Index): A goodness-of-fit index used to assess the fit between the model and observed data. NFI (Normed Fit Index): A normed fit index used to evaluate model fit. IFI (Incremental Fit Index): An incremental fit index used to assess the degree of improvement in model fit. AGFI (Adjusted Goodness-of-Fit Index): An adjusted goodness-of-fit index that considers model degrees of freedom, used to evaluate model fit. These five indicators should be > 0.9, with a higher value indicating better model fit. RMSEA (Root Mean Square Error of Approximation): A root mean square error of approximation used to evaluate model fit. An RMSEA < 0.05 indicates close model fit. χ^2^/DF (Chi-Square Divided by Degrees of Freedom): A ratio of chi-square value to degrees of freedom used to assess model goodness of fit. χ^2^/DF < 3 indicates close model fit.

Data were statistically analyzed using SPSS 24.0. The enumeration data were represented as case counts and percentages, and the measurement data that followed a normal distribution were statistically described. A chi-square test was used for statistical analysis of intergroup comparisons because the dependent variable was binary, and the independent variable was continuous. In addition, independent sample t-tests were used for analysis. Depressive symptoms were assessed through multiple linear regression analysis to analyze the influential factors. Subsequently, path analysis was employed to investigate the internal relationships among the variables. The model was analyzed using Amos 25.0, and a path analysis diagram was generated. The final path analysis model was achieved through continuous correction of the model fitting degree. Finally, a *P*-value of < 0.05 indicated that differences were statistically significant.

### Ethics approval and consent to participate

The study was reviewed and approved by the Ethics Committee of Affiliated Hospital of Nantong University (approval number: 2022-K150-01). All methods were performed in accordance with the relevant guidelines and regulations (Declaration of Helsinki). All participants have signed the Informed Consent.

## Results

### Characteristics of the study population

Of 1088, 1071 pregnant women met the inclusion criteria and were included in the final analysis. According to cutoff score of 10 of the EDPS, 208 (19.4%) had depressive, whereas 863 (80.6%) did not. The maternal education level (*P* = 0.026), paternal education level (*P* = 0.015), underlying disease (*P* = 0.050), currently working (*P* = 0.010), incontinence during pregnancy (*P* = 0.014), and hyperthyroidism (*P* < 0.001) were statistically significant between the depressive and non-depressive group (Table 1).

**Table 1:** Characteristics of the subjects enrolled in this study.

Variables	Non-AD	AD	*P*
	(n = 863)	(n = 208)	
Age			
< 30	443 (51.3%)	111 (53.4%)	0.598
≥ 30	420 (48.7%)	97 (46.6%)	
Educational level (oneself)			
Below bachelor	310 (35.9%)	92 (44.2%)	0.026
Bachelor or above	553 (64.1%)	116 (55.8%)	
Educational level (husband)			
Below bachelor	339 (39.3%)	101 (48.6%)	0.015
Bachelor or above	524 (60.7%)	107 (51.4%)	
Family monthly income (CNY ¥)			
< 5000	19 (2.2%)	10 (4.8%)	0.077
5000–10,000	276 (32.0%)	76 (36.5%)	
10,001–20,000	425 (49.2%)	94 (45.2%)	
> 20,000	143 (16.6%)	28 (13.5%)	
First marriage			
Yes	823 (95.4%)	195 (93.8%)	0.335
No	40 (4.6%)	13 (6.3%)	
Underlying disease			
Yes	41 (4.8%)	17 (8.2%)	0.050
No	822 (95.2%)	191 (91.8%)	
Pain			
Yes	55 (6.4%)	21 (10.1%)	0.060
No	808 (93.6%)	187 (89.9%)	
Gestational weeks			
32–35^+6^	624 (72.3%)	151 (72.6%)	0.933
36–40	239 (27.7%)	57 (27.4%)	
Abortion history			
No	608 (70.5%)	136 (65.4%)	0.486
Yes	255 (29.5%)	72 (34.6%)	
Parity			
Primipara	626 (72.5%)	149 (71.6%)	0.794
Multipara	237 (27.5%)	59 (28.4%)	
Assisted reproduction			
No	753(87.3%)	175 (84.1%)	0.235
Yes	110 (12.7%)	33 (15.9%)	
Residence			
Downtown	507 (58.7%)	116 (55.8%)	0.648
Town	219 (25.4%)	54 (26.0%)	
Village	137 (15.9%)	38 (18.3%)	
Complications			
No	669 (77.5%)	153 (73.6%)	0.225
Yes	194 (22.5%)	55 (26.4%)	
Working			
Yes	354 (41.0%)	65 (31.3%)	0.010
No	509 (59.0%)	143 (68.8%)	
Urinary incontinence			
Yes	375 (43.5%)	110 (52.9%)	0.014
No	488 (56.5%)	98 (47.1%)	
SARS-CoV-2 infection			
Yes	621 (72.0%)	151 (72.6%)	0.854
No	242 (28.0%)	57 (27.4%)	
Gestational hypertension			
Yes	13 (1.5%)	4 (1.9%)	0.666
No	850 (98.5%)	204 (98.1%)	
Hyperthyroidism during pregnancy			
Yes	6 (0.7%)	9 (4.3%)	< 0.001
No	857 (99.3%)	199 (95.7%)	
Gestational diabetes			
Yes	117 (13.6%)	36 (17.3%)	0.165
No	746 (86.4%)	172 (82.7%)	
Hypothyroidism in pregnancy			
Yes	52 (6.0%)	8 (3.8%)	0.220
No	811 (94.0%)	200 (96.2%)	

### Analysis of the influencing factors associated with AD in pregnant women

Linear regression analysis was used to analyze the influencing factors associated with AD in pregnant women. Factors with *P* < 0.05 in single-factor analyses (Table 2) were included in the multiple linear regression model (Table 3**)**. Higher pregnancy stress (β: 0.082, 95% confidence interval [CI] 0.062–0.102, *P* < 0.001), higher antenatal anxiety (β: 0.210, 95% CI 0.164–0.256, *P* < 0.001), poor sleep quality (β: 0.171, 95% CI 0.110–0.236, *P* < 0.001), and hyperthyroidism (β: 1.819, 95% CI 0.300–3.340, *P* = 0.019) were the influencing factors associated with increased risk of depressive in the pregnant women (β > 0, *P* < 0.05). However, better social support (β: − 0.028, 95% CI − 0.053 to − 0.006, *P* = 0.013), marital satisfaction (β: − 0.155, 95% CI − 0.193 to − 0.116, *P* < 0.001), and mental resilience (β: − 0.129, 95% CI − 0.176 to − 0.104, *P* < 0.001) were associated with decreased risk of depressive in the pregnant women (β < 0, *P* < 0.05). Postpartum anxiety had the greatest impact on postpartum depressive. Means, standard deviations, and the correlation of study variables are shown in Table 4**.**

**Table 2:** Results of univariate analysis of antenatal depression.

Variables	β	SE	95% CI		*P*
			Lower	Upper	
Educational level (oneself)	− 0.268	0.147	− 0.557	0.020	0.068
Educational level (husband)	− 0.451	0.144	− 0.732	− 0.169	0.002
Working	0.645	0.279	0.098	1.193	0.021
Urinary incontinence	− 1.017	0.273	− 1.552	− 0.482	＜0.001
Hyperthyroidism during pregnancy	3.326	1.158	1.053	5.598	0.004
PPS	0.226	0.010	0.206	0.246	＜0.001
DASS	0.522	0.024	0.475	0.569	＜0.001
SPPP	− 0.212	0.012	− 0.235	− 0.188	＜0.001
MSS	− 0.412	0.018	− 0.448	− 0.376	＜0.001
PSQI	0.569	0.040	0.491	0.647	＜0.001
CD-RISC-10	− 0.385	0.017	− 0.418	− 0.352	＜0.001

*EPDS* Edinburgh Postnatal Depression Scale, *DASS* Depression Anxiety Stress Scale, *PPS* Pregnancy Pressure Scale, *PSSS* Perceived Social Support Scale, *MSS* Marital Satisfaction Scale, *PSQI* Pittsburgh Sleep Quality Index, *CD-RISC-10* 10-item Connor-Davidson Resilience Scale.

**Table 3:** Results of multiple linear regression analysis on antenatal depressive.

Variables	β	SE	95% CI		*P*
			Lower	Upper	
PPS	0.082	0.010	0.062	0.102	< 0.001
DASS	0.210	0.023	0.162	0.256	< 0.001
SPPP	− 0.028	0.012	− 0.053	− 0.006	0.013
MSS	− 0.155	0.020	− 0.193	− 0.116	< 0.001
PSQI	0.171	0.032	0.110	0.236	< 0.001
CD-RISC-10	− 0.129	0.034	− 0.176	− 0.104	< 0.001
Hyperthyroidism	1.819	0.776	0.300	3.340	0.019

*EPDS* Edinburgh Postnatal Depression Scale, *DASS* Depression Anxiety Stress Scale, *PPS* Pregnancy Pressure Scale, *PSSS* Perceived Social Support Scale, *MSS* Marital Satisfaction Scale, *PSQI* Pittsburgh Sleep Quality Index, *CD-RISC-10* 10-item Connor-Davidson Resilience Scale.

**Table 4:** Means, standard deviations, and the correlation of study variables (n = 1071).

	Mean	SD	EPDS	DASS	PPS	PSSS	MSS	PSQI	CD-RISC-10
EPDS	5.21	4.490	1						
DASS	5.33	4.870	0.559*	1					
PPS	0.405	0.369	0.560*	0.514*	1				
PSSS	71.98	10.134	− 0.469*	− 0.316*	− 0.375*	1			
MSS	42.20	6.134	− 0.564*	− 0.353*	− 0.411*	0.546*	1		
PSQI	6.69	3.172	0.407*	0.416*	0.310*	− 0.190*	− 0.275*	1	
CD-RISC-10	28.19	6.666	− 0.574*	− 0.386*	− 0.433*	0.557*	− 0.562*	− 0.278*	1

*EPDS* Edinburgh Postnatal Depression Scale, *DASS* Depression Anxiety Stress Scale, *PPS* Pregnancy Pressure Scale, *PSSS* Perceived Social Support Scale, *MSS* Marital Satisfaction Scale, *PSQI* Pittsburgh Sleep Quality Index, *CD-RISC-10* 10-item Connor-Davidson Resilience Scale.

*P < 0.001.

### Path analysis of influencing factors for AD

**Figure 1:**
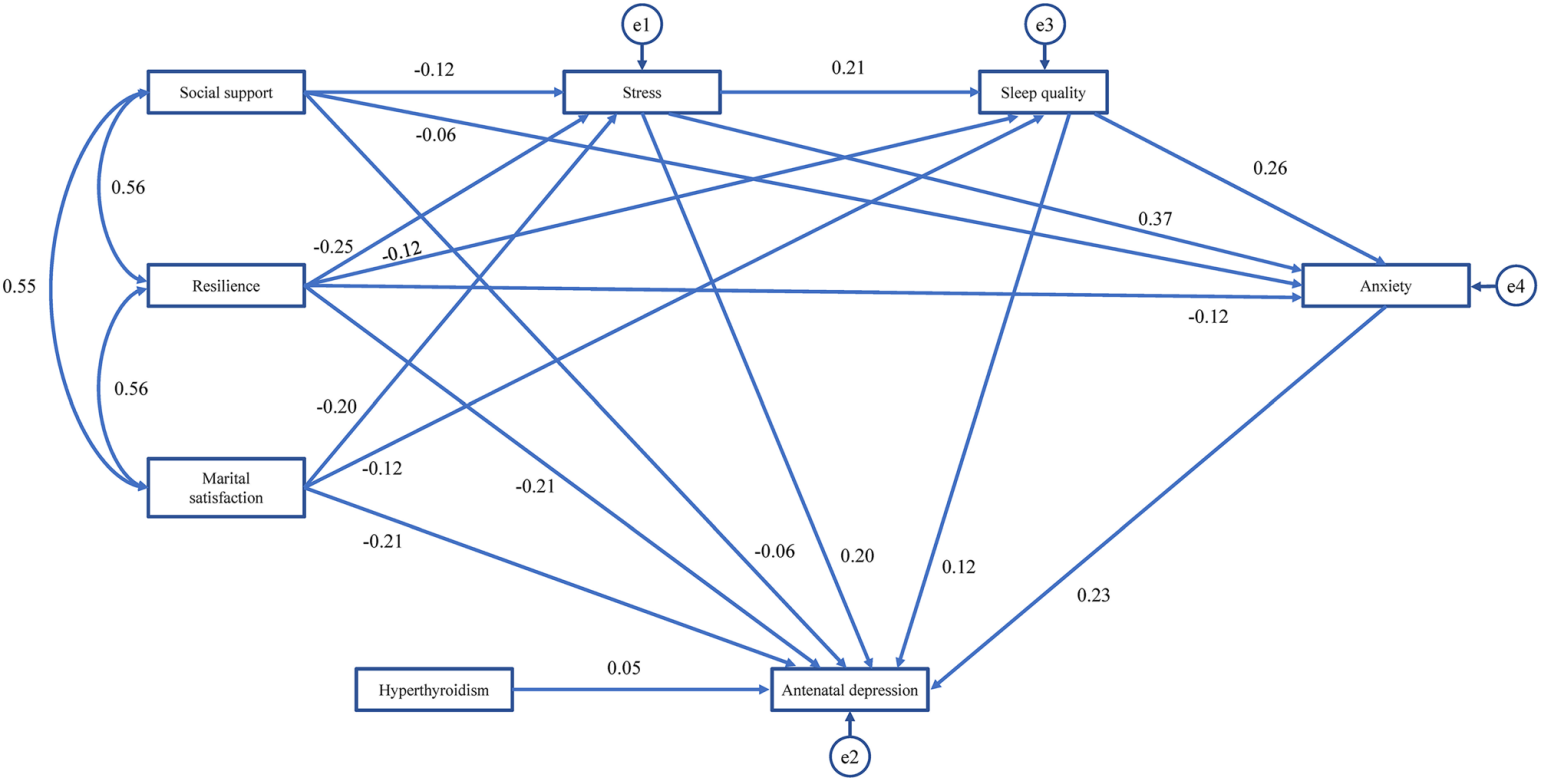
Path analysis of influencing factors for depressive of pregnancy.

The overall fitting effect of this model was as follows: CFI = 0.999, GFI = 0.998, NFI = 0.996, IFI = 0.999, AGFI = 0.990 (> 0.9), RMSEA = 0.015 (< 0.05), and χ^2^/DF = 1.238 (< 3), meeting the criteria. This indicates that the path analysis model has good fit (Table 5**)**. The model parameters demonstrated that the fitting effect of the model was acceptable^50^. The model was continuously revised by referring to the model fitting degree, and the influencing factors for AD were finally included in the path analysis. These included mental resilience, pregnancy stress, marital satisfaction, antenatal anxiety, sleep quality, social support, and hyperthyroidism. Table 6 represents the standardized coefficients of the path analysis. The variables in the paths are ranked in descending order of their total effects as follows: resilience (− 0.340) > stress (0.322) > marital satisfaction (− 0.300) > anxiety (0.230) > sleep quality (0.181) > social support (− 0.118) > hyperthyroidism (0.048). The lower and upper bounds of the 95% confidence interval (CI) values, which do not include 0, indicate that the paths are significant. In this study, all paths have a 95% CI that does not include 0, indicating that all paths are significant. All factors had both direct and indirect effects on AD but antenatal anxiety and hyperthyroidism only had direct effects. The path analysis is shown in Fig. 1.

**Table 5:** Goodness of fit indices for the model.

Index	CFI	GFI	AGFI	NFI	IFI	RMSEA	χ^2^/df
	0.999	0.998	0.990	0.996	0.999	0.015	1.238

*CFI* Comparative Fit Index, *GFI* Goodness of Fit Index, *AGFI* Adjusted Goodness of Fit Index, *NFI* Normed Fit Index, *IFI* Incremental Fit Index, *RMSEA* Root Mean Square Error of Approximation, *X2* Chi-square test df: degree of freedom.

**Table 6:** Standardized direct, indirect, and total effect of all study pathways (n = 1071).

Pathway	Direct effect β (95% CI)	Indirect effect β (95% CI)	Total effect β (95% CI)
DASS → EPDS	0.230 (0.169, 0.290)	–	0.230 (0.169, 0.290)
PPS → EPDS	0.203 (0.145, 0.270)	0.119 (0.090, 0.152)	0.322 (0.266, 0.384)
PSSS → EPDS	− 0.063 (− 0.122, − 0.007)	− 0.055 (− 0.088, − 0.026)	− 0.118 (− 0.186, − 0.052)
MSS → EPDS	− 0.212 (− 0.271, − 0.156)	− 0.088 (− 0.122, − 0.057)	− 0.300 (− 0.367, − 0.234)
PSQI → EPDS	0.122 (0.076, 0.168)	0.059 (0.041, 0.083)	0.181 (0.137, 0.228)
CD-RISC-10 → EPDS	− 0.210 (− 0.267, − 0.157)	− 0.130 (− 0.164, − 0.099)	− 0.340 (− 0.406, − 0.278)
Hyperthyroidism → EPDS	0.048 (0.012,0.087)	–	0.048(0.012,0.087)

*EPDS* Edinburgh Postnatal Depression Scale, *DASS* Depression Anxiety Stress Scale, *PPS* Pregnancy Pressure Scale, *PSSS* Perceived Social Support Scale, *MSS* Marital Satisfaction Scale, *PSQI* Pittsburgh Sleep Quality Index, *CD-RISC-10* 10-item Connor-Davidson Resilience Scale.

## Discussion

The detection rate of AD in this study was 19.4%, higher than the results of an international meta-analysis (11.9%)^2^. We found that higher pregnancy stress, higher antenatal anxiety, poor sleep quality, and hyperthyroidism were the influencing factors associated with an increased risk of AD in pregnant women. However, better social support, marital satisfaction, and mental resilience were associated with decreased risk of AD in pregnant women. The path analysis indicated that while all the factors had direct effects on AD, mental resilience had the biggest influence on AD. In addition to antenatal anxiety and hyperthyroidism, other factors have an indirect effect on AD. In path analysis, there is only a unidirectional logical relationship since AD is chosen as the outcome variable. Therefore, there can only be a unidirectional path from anxiety to AD. Path analysis reveals that resilience, social support, stress, and sleep quality not only have a direct impact on AD but also influence AD through the mediating effect of anxiety. Anxiety plays a mediating role in the above factors and AD, making it an important influencing factor.

More and more studies are exploring the link between AD and pregnant women, and their findings indicate that the rate of detecting prenatal psychological disorders was higher during the pregnancy period than during the puerperium period^14^. Reportedly, while there are various factors affecting AD, the factors also significantly differ across countries and even across regions within these countries. The findings of our study indicated that AD should be studied synthetically without neglecting any potential factors. Therefore, we constructed a path analysis model to determine the internal relationship between the different factors described above and how they influence AD.

According to the absolute values of the path coefficients, the factors influencing AD were ranked in descending order of their impact, as follows: mental resilience > pregnancy stress > marital satisfaction > prenatal anxiety > sleep quality > social support > hyperthyroidism. Mentally resilient pregnant women are more likely to better regulate their depressive. Women with high levels of resilience are flexible and capable of managing stress in a rational manner, thus achieving positive psychological outcomes when confronted with adversity. A study found that resilience acts as a protective factor for mental health^51^. Resilience empowers individuals to effectively combat the detrimental impacts of psychological challenges, such as depression, and enhances their capacity to uphold mental well-being. A previous study also confirmed that AD can be mitigated by the positive psychological traits of resilience^52^. Therefore, psychological counseling and therapeutic measures, such as stress management and resilience enhancement training, should be provided to pregnant women to reduce the negative impact of pregnancy on AD prevalence. Resilience can also affect pregnancy pressure, prenatal anxiety, and sleep during pregnancy^30,53,54^. Our path analysis suggested that resilience not only had a direct effect on depression during pregnancy but also had an indirect effect by influencing pregnancy pressure, prenatal anxiety, and sleep, thus explaining this complex relationship.

The second factor influencing the severity of AD is pregnancy stress, which refers to the psychological turmoil or threat caused by various stressful events and adverse factors during pregnancy. Pregnant women exposed to many sources of stress who are unable to adapt to these changes are at high risk of developing AD. In China, the prevalence of pregnancy stress is approximately 90%^7^. With the increasing social attention given to pregnant women’s mental health, family and social support toward them has improved, thus reducing the pregnancy pressure on pregnant women to a certain extent. Interestingly, however, most of the stresses arise from the pregnant women themselves, such as worrying about their baby’s health and not being able to effectively play the role of parents, which need to be managed by changing the pregnant woman’s thoughts. Stress caused by role change is the most important stressor of pregnancy stress, and pregnant women experience a gradual increase in fear of the unknown, from physical to role changes^55^, eventually causing severe pregnancy pressure. AD was positively correlated with pregnancy stress, suggesting that prenatal depressive symptoms worsened as pregnancy stress increased. Therefore, during pregnancy, pregnant women should maintain an optimistic attitude and relieve stress to reduce the risk of prepartum depression.

There exists an association between poor sleep quality, the state of stress during pregnancy, and AD condition^56^. According to the FinnBrain Birth Cohort Study^23^, the quality of sleep throughout pregnancy has a correlation with the emergence of depressive symptoms in the third trimester. Owing to the discomfort and substantial physical strain in the later stages of gestation, women often find it challenging to breath properly during slumber, and maintaining an uncomplicated flat resting position becomes difficult. Therefore, sleep disturbances have a high prevalence in the last trimester of pregnancy.

Furthermore, while many researchers found that stress, anxiety, and depressive symptoms coexisted^57,58^, only a few studies in China have discussed them collectively. Clark and Watson^59^ indicated an overlap between anxiety and depressive symptoms, thus explaining their coexistence. Our path analysis indicated that anxiety mediated the effects of prenatal stress and depression, clarifying the subtle relationship between them.

The third influence degree affecting AD symptoms was marital satisfaction. Higher marital satisfaction was a protective factor for prenatal depression, in line with previous research^60^. Similarly, several studies have shown that marital satisfaction is associated with mental health^61,62^. Perhaps, in a troubled marriage, the spouse fails to fulfill a supportive role in improving the emotional state^63^. As a result of inadequate support and ineffective communication, pregnant women in problematic marriages are more susceptible to AD. A Korean study also highlighted the significance of implementing marriage satisfaction counseling programs to prevent depression in pregnant women during the transition to motherhood^64,65^. Given the physical and psychological changes women undergo during pregnancy and the challenges/fear of transitioning to parenthood, poor relationships with their spouses would only worsen AD symptoms, further reducing the quality of their marital relationships^66^. These circumstances may give rise to psychological disorders in pregnant women, including depression^67^. Satisfaction with marital communication was a protective factor against pregnancy pressures^9^, which can reduce AD prevalence.

The multiple linear regression analysis indicated that while all factors had both direct and indirect effects on AD but antenatal anxiety and hyperthyroidism only had direct effects A study found that subclinical hyperthyroidism increases the risk of depression in pregnancy^68^. Hormonal changes during pregnancy may affect the thyroid, causing hyperthyroidism or exacerbating existing thyroid problems^69^. Subclinical hyperthyroidism was seen in 6% of women in the third trimester. In the third trimester, depressed women have significantly higher FT (4) concentrations, lower TSH concentrations, and higher prevalence of subclinical hyperthyroidism compared with non-depressed women. There is an association between thyroid dysfunction and depression in the third trimester of pregnancy. The clinical manifestations of abnormal thyroid hormone levels can manifest as various neurological or psychiatric changes, but sometimes the causal relationship is unclear. As pregnancy suppression may affect pregnancy outcomes, it is necessary to assess thyroid function during pregnancy^70^. By investigating the role of hyperthyroidism in AD, researchers can gain a better understanding of how thyroid function affects mood and mental health during pregnancy. This knowledge may contribute to the development of more effective screening methods, prevention strategies, and targeted interventions for individuals at risk of AD.

Prenatal anxiety only had a direct effect on AD symptoms; however, many factors can affect AD through prenatal anxiety. Reportedly, there is a high prevalence of prenatal anxiety among Chinese pregnant women, ranging from 7.9 to 68.4%^7,71^. Prenatal anxiety is positively correlated with AD^25^, and the AD predictors are prenatal anxiety, social support, resilience, pregnancy stress, and sleep quality^25,72,73^. Our path analysis indicated that resilience, pregnancy pressure, social support, and sleep quality not only directly affected AD but also had a mediating effect on anxiety. Although social support is generally seen as a protective factor against depression in pregnant women, the total correlation between pregnant women's depression and social support in this path analysis was found to be statistically insignificant. This finding is inconsistent with the results reported in a previous study^74^. It is possible that the reason for this inconsistency is the inadequate sample size of previous studies, resulting in a lack of representativeness. The absence of social support is another key element that has a strong association with heightened pregnancy pressure and prenatal anxiety. The provision of social support involves a dynamic interplay between the perceived support and actual support that individuals receive in various forms (such as informational, practical, and emotional support) from numerous sources, such as family, friends, neighbors, work associates, and participating groups^75^. Factors, such as the absence of support and care from individuals besides one's spouse, friends within the community, partners, and family members^76^, as well as the lack of support obtained by engaging in group activities, partially represent insufficient social support. These factors are linked to pregnancy pressure, symptoms of prenatal anxiety, and eventually AD.

Compared to previous studies, this research focuses on third trimester women in the Nantong region of China. The population and geographic area surveyed are different from previous studies. This study has incorporated variables related to AD, exploring the interrelationships among these variables and their impact on AD. This study may have employed path analysis, elucidating the nature, magnitude, and significance of path coefficients to better understand the relationships between variables in the model. Path coefficients represent the direct and indirect relationships between variables in structural equation modeling. By interpreting these path coefficients, we can understand how variables interact with each other and the extent to which they contribute to the overall explanatory power of the model. This study was conducted in three hospitals in the Nantong region, providing a larger statistical power and generalizability of findings. This study was conducted more recently, taking into account any changes or developments in the field, yielding new insights and understanding from previous research. Overall, these differences enhance our understanding and insights into AD by offering new perspectives, insights, and updated information.

We will consider the implications of our findings for healthcare professionals, such as obstetricians, gynecologists, and mental health providers. We will explore how our research can contribute to enhancing their understanding of AD and help shape their clinical decision-making, including the provision of appropriate support, counseling, and referral services. We will also focus on how to apply the relevant research findings of factors such as social support, resilience, marital satisfaction, sleep quality, stress, and anxiety in clinical settings. We will develop individualized intervention plans to provide effective support and treatment. By addressing these points, we aim to bridge the gap between our research findings and their practical applications in clinical settings.

This study investigated the relationships among factors influencing the symptoms of AD and provided an additional theoretical basis for clinical intervention. However, it has several limitations. First, although path analysis can be performed by estimating the direct and indirect effects of multiple variables in the model, we could not conclude that path analysis can fully test this complexity. Therefore, other analyses are needed to test and validate this complexity. Second, although there are many factors influencing AD, we could not determine whether all these factors also have an effect (direct or indirect) on AD. Further research is needed to validate our findings. Third, although the convenience sampling method provides the advantage of expedited results, especially the ability to distribute surveys widely and promptly, there is a potential nonrandomization selection bias, which may affect the generalizability of the results. Therefore, further studies are warranted to validate our results. Fourth, the cross-sectional nature of the study may limit the ability to draw causal inferences. Fifth, anxiety and depression share some overlapping symptoms. Including anxiety as a direct factor in a model primarily focused on depression might confound the results. To accurately interpret the relationships, it’s important to clearly define each construct and consider how they interact.

## Conclusion

Pregnancy stress, prenatal anxiety, social support, marital satisfaction, sleep quality, resilience, and hyperthyroidism have direct effects on AD. While antenatal anxiety and hyperthyroidism only had a direct effect on AD, resilience had the largest total effect on AD. This study can provide the basis for early screening and interventions to prevent the occurrence of AD.

## Data Availability

The raw data of the current study would be available from the corresponding author on reasonable request.
